# Words

**Published:** 1881-01

**Authors:** J. J. Keyes


### WORDS.

J. J. KEYES.

That heading does not quite express it. Perhaps the word "vocabulary" would have answered better; and yet, some people, on seeing it, would have thought right away of a pocket dictionary; and as dictionaries, whether large or small, are not usually considered interesting reading, they would have glanced at the heading and skipped the article. But, dear reader, let me tell you at once, that by "vocabulary," I should not have meant a dictionary, and that by "words" I do not mean a mere orderly assemblage of letters in pronounceable form. I wish to chat with you about three minutes, about the advantage of having a vocabulary of your own; of having a dictionary in your mind; of having a choice and varied collection of words treasured up in your mental storehouse, and always at your service. You have noticed, I presume, that an accomplished musician almost invariably has an extensive *repertoire*, and can sit down to his instrument and play a variety of tunes, or sing any number of songs. What this *repertoire* is to the musician, a good vocabulary is to a person in conversation, or in an attempt to express his ideas. You have seen musicians whom you have envied on account of the variety and brilliancy of their accomplishments. And so have you seen conversationalists, orators, writers and authors, who have excited both your envy and your admiration by the fluency of their language and the variety and richness of their diction. Words are the signs of ideas and the vehicles of thought; and if we would acquire facility and clearness in the expression of our ideas, we must make ourselves rich in words. Many people in trying to state their opinions, or relate an occurrence, or even in carrying on an ordinary conversation, often hesitate, or break down or make a bad jumble of it, for want of the right word, or words. The difficulty is that their vocabulary is limited. There are words in plenty; but they have not learned them. The ten thousand words in our language that are said to be in common use, furnish a vocabulary sufficiently extensive and varied to enable one to express almost any idea, and do it clearly. People who experience the difficulty of which I am speaking, should enlarge their *repertoire* of words. It is said of Daniel Webster, that during the early part of his professional life, he was a patient student of the dictionary, learning three words daily, and seeking an early opportunity to use them. In this manner, he acquired that rich vocabulary for which he was distinguished, and which enabled him to clothe his written and spoken utterances in such noble and appropriate diction. One reason why many preachers and other public speakers become at length monotonous and wearisome to their regular hearers, is because they fall into what we may call verbal ruts. They travel, in their methods of expression, in circles. They cling to certain pet words and phrases. Their acquaintance with words is limited, and they do not exert themselves to enlarge it. The mind likes variety, and insists upon new verbal combinations, and new methods and forms of expression, both in speaker and in author. It is no less desirable in a conversationalist. The man or woman whose vocabulary is extensive, and who surprises you in almost every sentence with some new word, or some old word used in new connections and a new sense, and who always has at his command the right word to express his idea, is always interesting in conversation, and is the envy of those who are less accomplished. Of course I understand that people differ from one another in the matter of endowment. Some seem to have the faculty of language, and many seem to lack it. Some people seem to have the "gift of gab," and to be born conversationalists; while others seem to have the gift of silence, and find it difficult to tell what they know. But while this may be true, it is reasonable to suppose that any person who has a clearly defined idea in his mind, can acquire the ability to express it, so far as human language can express it. For a person to say: "I know what I wish to say, but I cannot find words to express it"—and we very often hear it said—is not a sufficient excuse for his inability. Any person who will follow Mr. Webster's rule, and learn three new words each day from the dictionary, will at the end of ten years have a vocabulary of nearly eleven thousand words, and will be able to express his ideas with ease, fluency and propriety. We should not attribute to nature that which is due only to our neglect.

There is another advantage of this study and accumulation of words. Words are not mere things. They are instinct with life and thought. They are both the coverings and the expression of ideas. They have a history; and many words have a history which opens up to the student a wide and interesting field of scientific, literary and moral thought. A word may be an epitome of some great history, or some fascinating and instructive events in the career of some individual or in the life of some nation. Thus, the acquisition of words with their history and meaning, enlarges our information, renders us more intelligent, and adds constantly to our stock of ideas, while at the same time, as we have seen, it increases our power of expression. They give us new ideas and a new facility in giving them utterance, at one and the same time. Many people would have more of this facility if, in reading, they would stop to look up the meaning of every new and unfamiliar word they come to. But very few people do this. The average reader of history, or fiction, or of the magazines and newspapers hardly ever does it. He guesses at the meaning from the connection, or else persuades himself it is of no consequence; whereas, it is often the case that the words guessed at, or not understood, contain the key to the whole story, or situation.

I wish more attention were given to this subject of words in our schools. A great deal of attention is given to language; to moods, tenses, conjugations and grammatical construction; and this is well; it is essential; but not half enough attention is given to the subject of words—their derivation, history, analysis and definitions. And for this reason it happens that our schools often turn out upon the world a lot of educated dunces and dummies, who may have a vast amount of knowledge, but who have no power or but a very feeble power to communicate it or make it effective for the world's good.

Now I am aware that many people are wordy enough already. They can tell us all they know—and a little more. We often wish to goodness they would stop talking and "give us a rest." Words roll out of their mouth endlessly, like eggs out of a wizard's mouth; or run out like water from a perennial spring. This article is not for them. Let them tarry not, even so much as to look at it. They are already the terror of the world. Pass on, pass on, O ye endless talkers, and heed none of our suggestions. But I believe many can profit by what has here been written. For such it has been written. Only use moderately and kindly the facility you may acquire. Words are useful, but they are sometimes hurtful, and even fatal. Many a poor fellow has been driven to desperation by them. The saddest of all inscriptions on a man's tomb stone, would be; "Talked to Death."

